# Effect and mechanism of polyphenols containing *m*-dihydroxyl structure on 2-amino-1-methyl-6-phenylimidazole [4, 5-b] pyridine (PhIP) formation in chemical models and roast pork patties

**DOI:** 10.1016/j.fochx.2024.101672

**Published:** 2024-07-18

**Authors:** Hao Dong, Qi Chen, Yan Xu, Chao Li, Weidong Bai, Xiaofang Zeng, Qingping Wu, Huan Xu, Jinhua Deng

**Affiliations:** a Guangdong Province Key Laboratory for Green Processing of Natural Products and Product Safety, South China University of Technology, Guangzhou 510640, China; b College of Light Industry and Food Sciences, Zhongkai University of Agriculture and Engineering, Guangzhou 510225, China; c Guangdong Provincial Key Laboratory of Microbial Safety and Health, State Key Laboratory of Applied Microbiology Southern China, Key Laboratory of Microbiomics and Precision Application, Ministry of Agriculture and Rural Affairs, Institute of Microbiology, Guangdong Academy of Sciences, Guangzhou 510070, China; d Guangdong Huankai Microbiology Science & Technology Co., Ltd, Guangzhou 510700, China

**Keywords:** 2-Amino-1-methyl-6-phenylimidazo [4, 5-*b*] pyridine (PhIP), Polyphenols containing *m*-dihydroxyl structure, Generation pathway, Inhibition mechanism

## Abstract

2-amino-1-methyl-6-phenylimidazole [4, 5-*b*] pyridine (PhIP) is a prevalent heterocyclic amine (HAA) found in heated processed meat. This study investigated the inhibitory impact of eight different types of polyphenols containing *m*-dihydroxyl structure on PhIP formation through a chemical model system. The structure-activity relationship and potential sites of action of polyphenols containing *m*-dihydroxyl structure were also analyzed. Then, the mechanism of inhibiting PhIP formation by kaempferol, naringenin and quercetin was speculated by UPLC-MS. Results showed that 8 kinds of polyphenols containing *m*-dihydroxyl structure had significant (*P* < 0.05) inhibition on the formation of PhIP in the chemical model system in a dose-dependent manner. In addition, PhIP was most significantly inhibited by naringenin at the same concentration, followed by kaempferol and quercetin (83.27%, 80.81% and 79.26%, respectively). UPLC-MS results speculated that kaempferol, naringenin, and quercetin formed a new admixture via an electrophilic aromatic substitution reaction with the intermediate product phenylacetaldehyde, preventing the formation of PhIP.

## Highlights

• Structure-activity of *m*-dihydroxyl polyphenols on PhIP formation was revealed.
• Key intermediates and possible adducts were identified by UPLC-MS.
• Polyphenol with *m*-dihydroxyl eliminated PhIP production in a dose-dependent manner.
• Electrophilic aromatic substitution reaction with polyphenols prevented PhIP formation.

## 1 Introduction

2-amino-1-methyl-6-phenylimidazole [4, 5-*b*] pyridine (PhIP) is a prevalent heterocyclic amine (HAA) found in heated processed meat, characterized by its relatively high concentration (Dong et al., 2020, Jiang et al., 2022). Studies have shown that the intake of PhIP is closely related to the development of cancer, and PhIP is classified as a group 2B carcinogen (Chen, Xu, Dong, Bai, & Zeng, 2024, Dong, Xian, Li, Bai, & Zeng, 2020, Lin, Lai, Nagabhushanam, Ho and Pan, 2022, Xu et al., 2021). The pathway of PhIP formation is illustrated in Fig. S1 (A). The phenylalanine degradation follows the Strecker reaction, wherein the resulting carbonyl-muscle is generated via an aldol condensation reaction involving the creatinine-anhydride adduct. When C-5 of creatinine and phenylacetaldehyde are nucleophilically added, 2-amino-1-methyl-5-(1-hydroxy-2-phenylethyl)-imidazole-4-ketone is produced. Following the formation of 2-amino-1-methyl-5-(2-propenylphenyl)-imidazole-4-ketone via dehydration, ammonia and formaldehyde contribute to the PhIP imidazole ring (Hidalgo, Navarro, & Zamora, 2018). In addition, the principal precursors (phenylalanine and creatinine) of PhIP formation were demonstrated by the ^13^C experiment of the phenylalanine model system. The molecular structure of phenylalanine requires a complete transfer of the C-3, amino nitrogen, and benzene ring to PhIP molecules. In a similar manner, PhIP molecules also incorporate the methyl carbon, 1-N, and amino nitrogen from creatinine (Zamora, Alcón, & Hidalgo, 2013). Thus, phenylalanine's carbon atoms combine to form the pyridine component of PhIP, whereas creatinine contributes to forming part of imidazole.

At present, the mitigation of PhIP has attracted the interest of a considerable number of relevant scholars. Adding spices or plant extracts rich in natural antioxidants is currently one of the most effective methods for inhibiting the production of PhIP. For example, rosemary, turmeric, and garlic added to chicken wings and pork belly have been shown to inhibit 11 HAAs formation (Kwon et al., 2023). Furthermore, a novel inhibitor known as Chinese chive was found to impede PhIP formation through the direct elimination of its critical intermediate, phenylacetaldehyde (Wang et al., 2021). These investigations indicated that spices and plant extracts containing polyphenols are the most essential components in inhibiting the production of PhIP. However, most of these studies only investigated the inhibitory effect of polyphenols on PhIP, and its inhibitory mechanism was rarely studied. Several researches have revealed that polyphenols could hinder the formation of PhIP by inhibiting its precursors, such as creatinine and phenylalanine (Cheng et al., 2023). Furthermore, research also indicated that polyphenols could inhibit the formation of PhIP through persistent adduction with phenylacetaldehyde (Fig. S1 (B)), which is the primary intermediary of PhIP (Zhou et al., 2018). In addition, some scholars have employed chemical model systems and molecular simulation approaches to investigate and found a certain association between the structural characteristics of polyphenols and their inhibition effect on PhIP generation. It was discovered that phenolic compounds with two hydroxyl structures on the meta site of the aromatic nucleus had a remarkable inhibition effect on PhIP formation (Salazar, Arámbula-Villa, Hidalgo, & Zamora, 2014). To be specific, the order of inhibitory effects on PhIP was as follows: *m*-dihydroxyl, *o*-dihydroxyl and *p*-dihydroxyl. Therefore, it was speculated that the polyphenol containing *m*-dihydroxyl structure inhibited PhIP synthesis more effectively than the polyphenol containing *o*-dihydroxyl and *p*-dihydroxyl structure. However, the structure-activity relationship of the inhibition of polyphenols containing *m*-dihydroxyl structures on PhIP formation remains to be further studied.

This study employed a chemical model system to investigate the inhibitory impact of eight different polyphenols containing *m*-dihydroxyl structure on the formation of PhIP. In addition, the structure-activity relationship of these polyphenols on PhIP formation was revealed as the mechanism of inhibition of these polyphenols on PhIP formation and the relationship between molecular structure and inhibitory effect. Furthermore, the essential intermediates and possible adducts were discovered using UPLC-MS technology, and the molecular mechanism of the three polyphenols containing *m*-dihydroxyl structure (kaempferol, naringenin, and quercetin) that inhibit PhIP formation was inferred. This study could serve as a scientific, theoretical, and methodological reference for controlling the number of HAAs in hot processed meat products and applying natural extracts rich in polyphenols. At the same time, it promotes human health and ensures the safety of meat consumption, both of which are practical implications of critical importance.

## 2 Materials and methods

### 2.1 Chemicals and reagents

98% purity of resveratrol, myricetin, galangin, apigenin, luteolin, kaempferol, naringenin, and quercetin were obtained from Ciyuan Biotechnology (Shaanxi, China). PhIP standard (99.9%) was acquired from Toronto Research Chemicals (North York, Ontario, Canada). Glucose, creatinine, creatine, phenylalanine, and phenylacetaldehyde were acquired from Yuanye Biotechnology (Shanghai, China), of which the purity is over 98%. Methanol, acetonitrile, and formic acid, with a purity of 99.8%, were supplied by Sinopharm Chemical Reagent Co., Ltd. (Shanghai, China).

### 2.2 Establishment of chemical model system

The establishment of chemical model systems is informed by the previously published laboratory paper (Xu et al., 2023). A 20 mL solution of diethylene glycol, with a water content of 50%, was injected into a reaction that contained glucose (0.2 mmol), phenylalanine (0.4 mmol), creatine (0.4 mmol), and creatinine (0.4 mmol). Following the addition of 0.1–0.5 mg/mL polyphenols (resveratrol, naringenin, apigenin, luteolin, myricetin, kaempferol, galangin, and quercetin) to the chemical system. Then, it is heated at 220 °C for 180 min. The model system experiment was carried out in triplicate. The inhibition rate of polyphenols on PhIP (inhibition rate %) is calculated as follows:

$$\text{inhibition}\;\text{rate}\%=\left(1-\frac{\text{the}\;\text{PhIP}\;\text{content}\;\text{of}\;\text{sample}}{\text{the}\;\text{PhIP}\;\text{content}\;\text{of}\;\text{control}}\right)\times 100$$

### 2.3 Preparation of roast pork patty

The pork was sourced from Carrefour supermarket (Guangzhou, China). After removing the visible fascia from the sample meat, put it into a meat grinder to make minced meat. After mixing well, 40 ± 0.1 g minced meat was weighed to make a round meat patty. Then, different concentrations of polyphenols (kaempferol, quercetin, naringenin) were added to the patty. A circular patty of 1 cm thick and 6 cm diameter is formed and placed in a jar. The patty was then roasted in an electric oven at 225 °C for 10 min per side.

### 2.4 Detection conditions

By utilizing HPLC-Q-Orbitrap HRMS, the concentrations of PhIP, precursors (creatinine, creatine, phenylalanine, and glucose), intermediates (formaldehyde and acetaldehyde), and creatinine were identified. The detection conditions were referred to our previously published paper (Xu et al., 2023).

By utilizing GC–MS, the concentrations of phenylacetaldehyde were identified. The detection conditions were referred to our previously published paper (Xu et al., 2023).

By utilizing Waters ACQUITY UPLC System (Milford, MA, USA) fitted with ACQUITY UPLC BEH C18 column (2.1 × 100 mm, 1.7 μm), the key intermediates and potential adducts were identified. Mobile phase A: 125 mM Ammonium hydroxide, mobile phase B: Methanol. The temperature of the column, the flow rate, and the sample injection volume were 35 °C, 2 μL, and 0.3 mL/min, respectively. The procedures for gradient elution were cited in the prior study (Xu et al., 2023). The ESI-MS operating conditions were referred to by Yang et al. (Yang, Blecker, Liu, Zhang, & Wang, 2023).

### 2.5 Statistical analysis

The statistical software SPSS 21.0 (IBM Corp., New York, USA) was utilized for a one-way ANOVA, and Duncan's procedure was employed for multiple comparisons.

## 3 Results and discussion

### 3.1 Effect of polyphenols containing m-dihydroxyl structure on PhIP and related substances

**Fig. 1:**
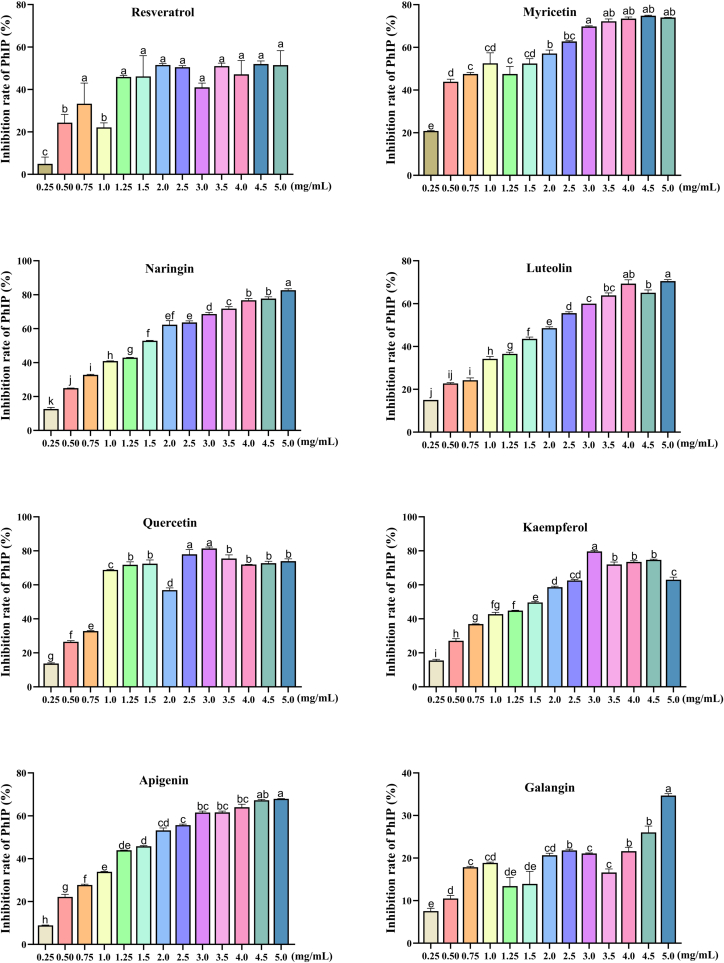
Effect of different concentrations of polyphenol containing *m*-dihydroxyl structure on PhIP formation in chemical model system (*n* = 3). Significant differences (*P* < 0.05) are shown by different letters above the error bars.

As can be seen from Fig. 1, the effects of 8 kinds of polyphenols containing *m*-dihydroxyl structure on PhIP formation were significantly different (*P* < 0.05). To be specific, following the addition of polyphenols, the inhibition rate of PhIP essentially increased in the concentration (0–1.0 mg/mL). However, the introduction of resveratrol resulted in an initial escalation followed by a subsequent decline in PhIP inhibition, and the highest rate of inhibition peaked at 2.0 mg/mL. Following the addition of galangin, the inhibition rate displayed instability, with the maximum inhibition rate observed at 5.0 mg/mL concentration. Compared with resveratrol and galangin, the inhibition rate of the other polyphenols containing *m*-dihydroxyl structure was relatively stable, and there was a downward trend at some intermediate concentrations, but this decline was not obvious in most cases. Among them, the contents of phenylalanine, PhIP, phenylacetaldehyde and acetaldehyde were dose-dependent with the added concentrations of naringenin, kaempferol and quercetin (*P* < 0.05). It indicated that kaempferol, quercetin and naringenin had good trapping effect on these compounds. In addition, the lowest concentrations of creatine and creatinine suggest that naringenin has the most effective capturing effect on them at a concentration of 0.75%. In conclusion, the inhibition rate of PhIP was positively correlated with the 8 kinds of polyphenols containing *m*-dihydroxyl structure. Furthermore, the inhibitory effect of all analyzed PhIP compounds exhibited a consistent pattern, whereby the addition of increased concentrations of polyphenols containing *m*-dihydroxyl structure resulted in a proportional increase in the inhibition rate until reaching a stable level. This conclusion is similar to the results of Xue et al. (Xue et al., 2022). The inhibitory rates of kaempferol on free and bound HAAs were 72.78% and 38.73%, respectively. The highest inhibitory rates of myricetin, luteolin, apigenin, resveratrol, and galangin on PhIP were 74.59%, 70.58%, 67.85%, 56.29% and 34.35%, respectively. In contrast to the findings of this study, some researchers (Zhao Lei, Yubin, et al., 2019) examined the effects of 15 polyphenols on the contents of 5 different kinds of HAAs in roast chicken breast and discovered that luteolin and naringenin had lower inhibitory rates. However, quercetin, apigenin, kaempferol, and myricetin had promoting effects on the formation of PhIP, which is contrary to the conclusion of this study. This could be caused by variations in the kind of raw meat, the method of preparation, and the dosage of polyphenols chosen.

### 3.2 Structure-activity relationship between polyphenols containing m-dihydroxyl structure and inhibition of PhIP formation

**Fig. 2:**
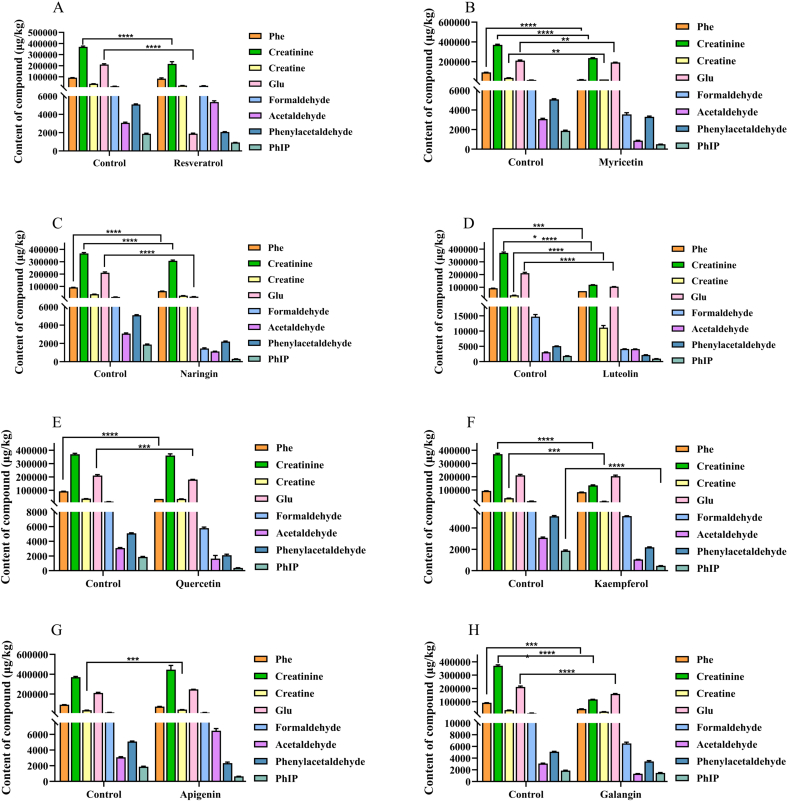
Content of PhIP, precursors and intermediates changed by the eight polyphenols containing *m*-dihydroxyl structure in chemical model system at 200 °C for 200 min. Profiles of resveratrol (4.5 mg/mL), myricetin (4.5 mg/mL), naringin (5.0 mg/mL), luteolin (5.0 mg/mL), quercetin (3.0 mg/mL), kaempferol (3.0 mg/mL), apigenin (5.0 mg/mL), and galangin (5.0 mg/mL) in the chemistry model system.

In order to further investigate the impact of polyphenols containing *m*-dihydroxyl structure on PhIP, these 8 kinds of polyphenols containing *m*-dihydroxyl structure were added to the chemical model system, and the contents of precursors, intermediates, and PhIP were compared with the control samples. As shown in Fig. 2, the contents of precursors, intermediates, and PhIP were generally reduced. Among them, the concentration of formaldehyde and acetaldehyde enhanced with resveratrol is greater than that of the control sample, indicating that resveratrol has no trapping effect on formaldehyde and acetaldehyde but rather functions as a promoter compared with other compounds. A similar result was obtained by adding apigenin. Moreover, the amounts of PhIP, precursors, and intermediates were drastically decreased. The effects of these polyphenols containing *m*-dihydroxyl structure on PhIP inhibition rate are shown in Table 1. For non-flavonoid phenolic compound, the inhibition rate of resveratrol on PhIP was 56.29%. The structure of resveratrol is different from the other seven polyphenols because it doesn't have a C ring. Instead, it has 7-OH in the A ring and 4’-OH in the B ring. Because of the difference of its structural formula, its inhibition rate is low. In addition, due to its *m*-dihydroxyl structure, resveratrol possesses a comparatively feeble capacity to eliminate free radicals. A correlation has been established in prior research between the rates of HAA inhibition and the activity of ABTS or DPPH. Specifically, a positive correlation has been observed between the clearance of ABTS or DPPH by polyphenols comprising solely *m*-dihydroxyl and the rates of HAA inhibition (Yang et al., 2023). This indicates that polyphenols containing *m*-dihydroxyl structure polyphenols serve as a highly effective structural foundation for suppressing the development of PhIP. However, its potent ability to eliminate PhIP suggests that scavenging free radicals may be a secondary mechanism rather than the main means of suppressing PhIP.

**Table 1:** Inhibition rate of hydroxyl positions and quantities of polyphenols containing *m*-dihydroxy structure in chemical model system (n = 3).

Polyphenols containing *m*-dihydroxyl structure		Chemical structure formula	CAS	Type	name	Number of hydroxyl groups	A-ring	B-ring	C-ring	Inhibition rate of PhIP(%)
Polyphenols	Resveratrol		501–36-0	Non-flavonoid phenolic compounds	5,7,4 ‘-trihydroxy-non-flavonoid phenol	3	5，7	4’	/	56.29%
	Galangin		548–83-4	Flavonol	5,7, 3-trihydroxy flavonol	3	5，7	/	3	34.35%
	Kaempferol		520–18-3	Flavonol	3,5,7,4 ‘-tetrahydroxy flavonol	4	5，7	4’	3	79.26%
	Quercetin		117–39-5	Flavonol	3,5,7,3 ‘,4 ‘-pentahydroxy flavonols	5	5，7	4′，5’	3	80.81%
	Myricetin		529–44-2	Flavonol	3,5,7,3 ‘,4 ‘,5 ‘-hexahydroxy flavonols	6	5，7	3′，4′，5’	3	74.59%
	Apigenin		520–36-5	Flavone	5,7,4 ‘-trihydroxy flavone	3	5，7	4’	/	67.85%
	Naringin		10,236–47-2	Flavone	5,7,4 ‘-trihydroxy flavone	3	5，7	4’	/	83.27%
	Luteolin		491–70-3	Flavone	5,7,3 ‘,4 ‘-tetrahydroxy flavone	4	5，7	3′，4’	/	70.58%

All hydroxyl groups of phenolic compounds are known to be situated in the meta site. Therefore, two components of the *m*-dihydroxyl structure difference that affect PhIP inhibition can be further investigated: the variation in the number and location of phenol hydroxyl group and alcohol hydroxyl group. For flavones, the inhibition rates of apigenin, naringenin, and luteolin on PhIP were 67.85%, 83.27%, and 70.58%, respectively. It was found that luteolin with a dihydroxyl group in the B ring and kaempferol with an *m*-hydroxyl group in the C ring were better at preventing PhIP formation than apigenin, which has an *m*-hydroxyl group in the B ring but no hydroxyl group in the C ring (Zhang et al., 2021; Zhao et al., 2020). Furthermore, it is worth noting that apigenin and naringenin share a common binding site for the A and B ring hydroxyl groups. The only distinguishing factor between them is the carbon bond on the C ring (double bond and single bond). This structural difference in the *m*-dihydroxyl group can lead to varying inhibition rates. At the same time, intramolecular hydrogen bonds are established between 4’-OH and 3’-OH, thereby enhancing the stability of the free radicals of 3′, 4′-o-dihydroxypolyphenol (Aakeröy, Epa, Forbes, Schultheiss, & Desper, 2013). In conclusion, inhibiting apigenin and luteolin on PhIP production may be related to its antioxidant activity. Furthermore, a hydroxyl group on flavonoids may impede the nucleophilic addition of phenylacetaldehyde, which is the precursor of PhIP, via steric hindrance.

For flavonols, the inhibition rates of quercetin, galangin, kaempferol, and myricetin on PhIP were 80.81%, 34.35%, 79.26%, and 74.59%, respectively. In the structural formula of galangin, there is no 4’-OH in the B ring. However, the other 7 kinds of polyphenols containing *m*-dihydroxyl structure have this hydroxyl group, and their inhibition rate is higher than that of galangin. This may be attributable to the absence of a hydroxyl group at the 4′-hydroxyl position in the B ring, which lacks a hydroxyl group, resulting in little inhibitory activity. It can be inferred that the importance of hydroxyl binding in the B ring (Ma et al., 2019). In addition, the carbon charge of myricetin demonstrates a significant electron density in specific carbons (C_6_ and C_8_), facilitating its interaction with carbonyl compounds through electrical substitution (Zhou et al., 2018). However, introducing extra hydroxyl and amino groups predominantly mitigates the inhibitory impact. As shown in Table 1, myricetin's inhibitory effect on PhIP is diminished in comparison to kaempferol and quercetin, which share the same structural characteristics due to the introduction of 5’-OH in the B ring. It is possible to deduce that adding an o-dihydroxyl group can augment the inhibitory effect of PhIP. Thus, it is hypothesized that the concurrent existence of 5-OH and 7-OH in the A ring, along with 4’-OH in the B ring, play a vital role in effectively preventing HAAs formation by polyphenols in meat. Conversely, the presence of 3-OH in the C ring diminishes the inhibitory effect of polyphenols on HAAs formation (Kim & Hur, 2018). On the other hand, the inhibition rate of naringenin on PhIP was higher than quercetin and kaempferol. This phenomenon could be attributed to the 4′-hydroxyl group in the B ring of the flavonoid. This group extends the flavonoid molecule's binding system, causing the entire molecule's electron cloud to be out of range. Consequently, the flavonoid could generate rather stable free radical intermediates (Teng et al., 2023). In conclusion, the inhibition rate of PhIP and the number of hydroxyl groups were irregular. Consequently, the inhibitory effect of polyphenols containing *m*-dihydroxyl structure on PhIP might be contingent on the location of hydroxyl groups within their structure than the number of hydroxyl groups.

Based on the above, it can be seen that quercetin containing catechol structure has the highest inhibition rate in flavonol structure, reaching 80.81%. In addition, the inhibitory rate of luteolin, which also contains catechol structure, was 70.58%. Prior research has demonstrated that polyphenols with catechol, resorcinol (such as orcinol, 3, 5-dihydroxypentylbenzene), resorcinol, hydroquinone, and hydroquinone exhibit superior inhibitory rates compared to polyphenols with other structures (Zamora & Hidalgo, 2016; Zamora & Hidalgo, 2020). This finding aligns with the result reported in this study. Although resveratrol has a resorcinol structure, polyphenols with a resorcinol structure exhibit substantially less inhibition than those with a catechol structure. This high inhibition may be related to its structure. As previously mentioned, phenolic compounds with hydroxyl groups at the meta site exhibit the highest carbonyl trapping capacity due to the electrophilicity of the aromatic carbon and hydroxyl groups of catechol (Zamora et al., 2013; Zamora & Hidalgo, 2016). This is because catechol has a strong electrophilicity due to the two hydroxyl groups at the ortho-site, which allows it to partially and rapidly bind to the active carbonyl group.

### 3.3 Effects of kaempferol, quercetin, naringenin on PhIP and related substances in roast pork patties

**Fig. 3:**
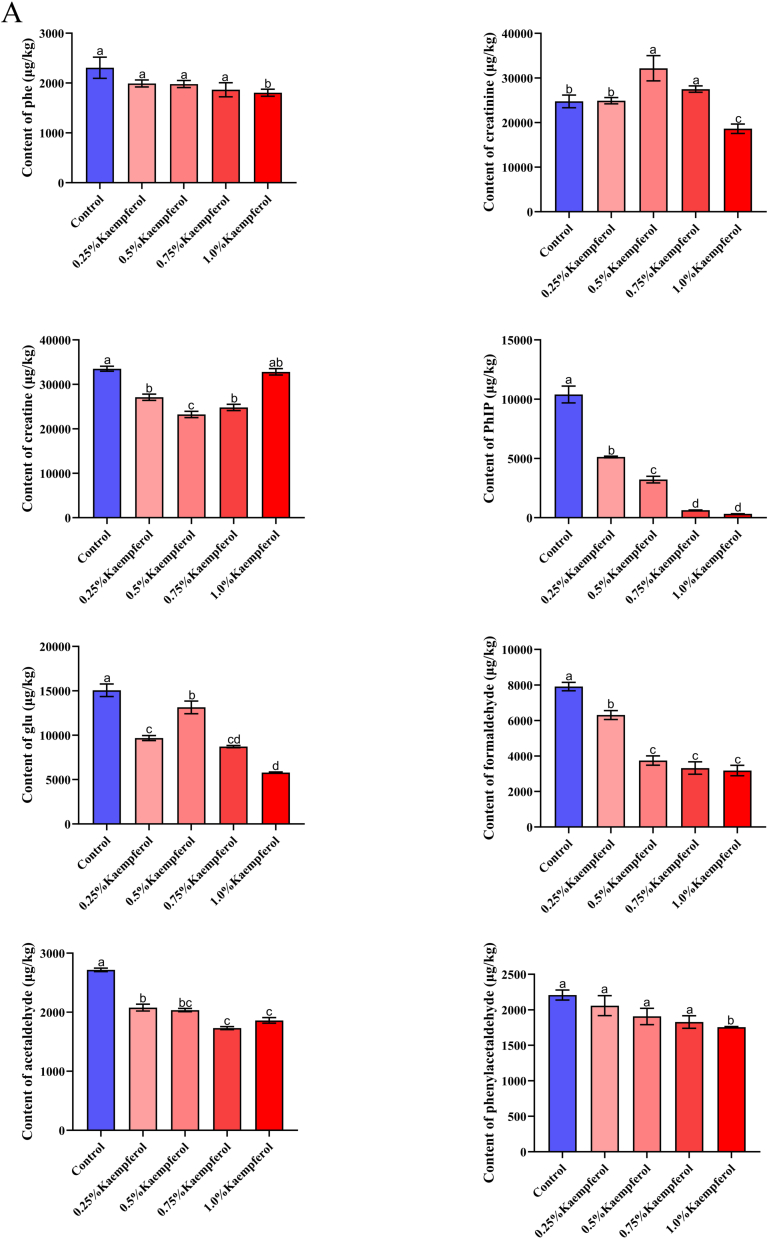

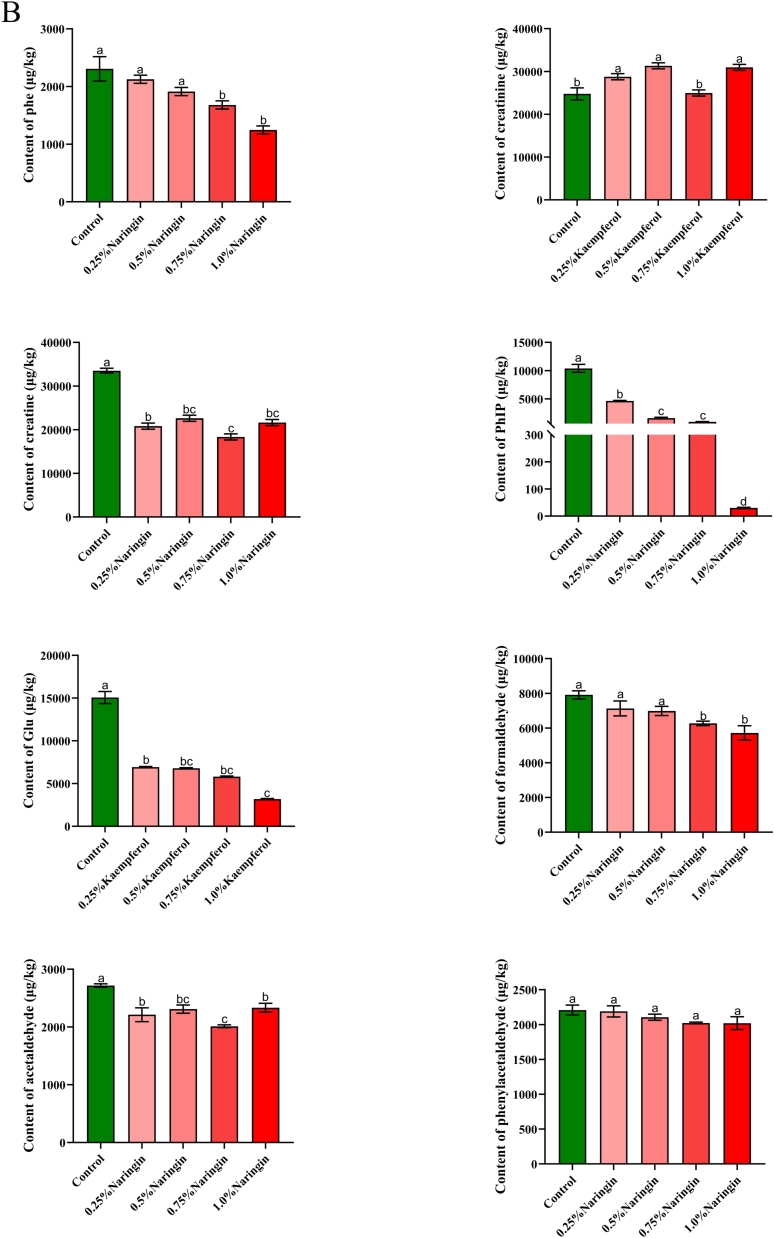

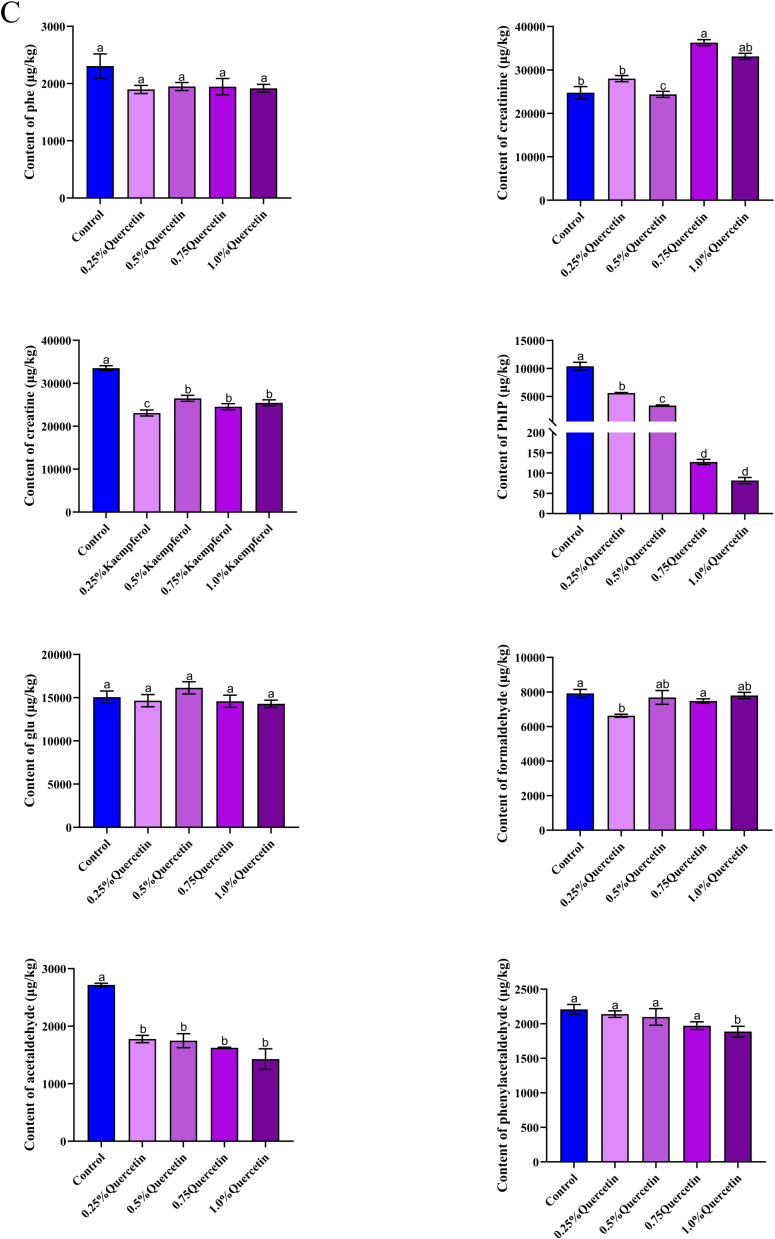
Effect of different contents of kaempferol (A), naringin (B), quercetin (C) on the content of phenylalanine, creatinine, creatine, PhIP, glucose, formaldehyde, acetaldehyde and phenylacetaldehyde in roast pork patties, respectively. Results with varying lowercase letters in the same series exhibit significant differences (*P* < 0.05).

Kaempferol, quercetin, and naringenin have the most potent inhibitory effect on PhIP among the eight types of *m*-dihydroxyl-structure polyphenols containing listed above. In order to further elucidate the difference between these three polyphenols, different concentrations of polyphenols were added to study their effects on PhIP and related substances (Fig. 3). Kaempferol, a common spice, mostly exists in medicinal plants. In addition to significantly enhancing the flavor of food, it possesses a powerful capacity to scavenge free radicals (Xue et al., 2022). As shown in Fig. 3 (A), the contents of phenylalanine, phenylacetaldehyde, and PhIP were dose-dependent with the added concentration of kaempferol. There was an initial decrease in creatine content to 0.5% as kaempferol content increased, followed by a subsequent increase (*P* < 0.05). The content of kaempferol in 0.25%–0.75% was higher than that in the control sample. Moreover, creatinine increased initially and subsequently decreased in response to increased kaempferol concentration. However, creatine and creatinine showed an opposite trend, which was consistent with the results of creatine dehydration cyclization into creatinine (Kreider, Jäger, & Purpura, 2022). Kaempferol also has a capturing effect on formaldehyde and acetaldehyde, as their levels progressively drop with increasing concentrations of kaempferol. In contrast to other substances, the concentration of glucose in samples supplemented with kaempferol was found to be lower than that of the control samples. To be more specific, the changing trend is that the concentration of kaempferol increased to 0.5%, and when the concentration of kaempferol reached a certain content, the content of glucose would decrease. This is because different sugars (rhamnose, glucose, and galactose) can combine with kaempferol to produce the glycoside form of kaempferol, which has a strong oxidation inhibition ability (Devi et al., 2015). Researchers Xue et al. (Xue et al., 2022) found that different concentrations of kaempferol (0.005%, 0.010%, 0.015%) resulted in a dose-dependent decrease in HAAs, which is similar to the changing trend in this study.

Naringenin is abundantly present in citrus fruits and tomatoes. This is a bioactive flavonoid that has a significant impact on human health. As shown in Fig. 3 (B), phenylalanine, PhIP, glucose, formaldehyde, and acetaldehyde contents were dose-dependent with the added concentration of naringenin (*P* < 0.05), suggesting that naringenin effectively captured these compounds. With the increase of naringenin concentration, creatine and creatinine contents showed the same change trend (*P* < 0.05), but creatine content was noticeably decreased compared to the control sample. However, creatinine content was higher than that of the control sample, consistent with the results of creatine dehydration cyclization into creatinine in the pork patty with kaempferol added above. In addition, naringenin with different concentrations had a dose-dependent effect on phenylacetaldehyde but had no statistically significant influence on phenylacetaldehyde content (*P* > 0.05). The minor molecule 6-C-(*E*-) phenylacetaldehyde) naringenin (6-CEPN) is produced when naringenin reacts with phenylacetaldehyde. Moreover, 6-CEPN could inhibit the development of human liver cancer cells and lung metastasis (Kang et al., 2019; Zhou et al., 2022). At 0.75% concentration, creatine and creatinine are the lowest points, indicating that naringenin has a good trapping effect on them. Moreover, the inhibition rate of 1.0% naringenin on PhIP was 99.7%. Previous studies have shown that naringenin has the highest efficacy against PhIP in both the chemical model system and the fried beef patty (Zhang et al., 2021), which aligns with the findings of this study. However, the association between the Trolox-equivalent antioxidant capacity and the effect on PhIP generation was weak, indicating that the scavenging of free radicals may not be the main mechanism of its intervention. (Li et al., 2023). In addition, the synergistic effect of phosphorylated κ-carrageenan (P-KC) and naringenin on HAA formation has been observed in recent studies to be more pronounced compared to the effects of P-KC or naringenin alone (Xue et al., 2023). This finding implied that a novel approach to inhibiting HAA may involve the combination of hydrophilic colloid and flavonoid.

Quercetin, one of the most commonly consumed flavonoids through diet, is a member of the flavonol family (Ulusoy & Sanlier, 2020). As shown in Fig. 3 (C), the contents of phenylalanine, PhIP, phenylacetaldehyde, and acetaldehyde were dose-dependent with quercetin. There was no significant difference in phenylalanine, and an important distinction was observed between the phenylacetaldehyde control sample and the sample supplemented with 1.0% quercetin (*P* < 0.05). Among them, the changing trend for creatine and creatinine was identical to kaempferol and naringenin following the addition of quercetin. The trend of glucose change following quercetin addition was identical to kaempferol. In addition, the rate at which quercetin inhibits PhIP is greater than that of kaempferol. This may be ascribed to the 3-hydroxy group that is present in kaempferol. In contrast, quercetin possesses 3 and 5–OH groups in its C and A rings, respectively, which are capable of supplying the greatest number of electrons (Jan et al., 2022). Studies have shown that quercetin, the main antioxidant of tomatoes, has the ability to impede the creation of PhIP. The highest level of inhibition seen is 67% at 10 mg/kg (Fan et al., 2018), among which quercetin also has a trapping effect on precursors and intermediates. In this study, the maximum inhibition degree of 1.0% quercetin was 99.21%. It can be seen that the generation of PhIP could be inhibited by removing these precursors and intermediates. However, polyphenol compounds continue to be restricted in their application to food products because of their potential adverse effects on the texture and flavor.

### 3.4 Inhibition mechanism of kaempferol, quercetin and naringenin on PhIP formation

Prior to investigating their inhibitory mechanism, the thermal stability of kaempferol, naringenin, and quercetin in chemical modeling systems was examined (Fig. S2 (A)). This is because the majority of polyphenols are prone to heat breakdown in a water-based solution (Kellil, Grigorakis, Loupassaki, & Makris, 2021). As can be seen from the Fig. S2 (B-G), 3 kinds of polyphenols containing *m*-dihydroxyl structure, four precursors, and diethylene glycol were not displayed in the signal data of the detector of the diode array of high-performance liquid chromatography, indicating that creatine and phenylacetaldehyde were degraded and transformed. At the same time, creatinine, diethylene glycol, phenylalanine, and glucose can also be observed on the chromatogram, and all show relatively good thermal stability. According to previous studies, the peak shown in Fig. S2 is mainly related to the A-ring part (Teng et al., 2023). Therefore, UPLC and MS are required to determine the structure, retention time, ultraviolet absorption spectrometry, and MS of reaction products.

**Fig. 4:**
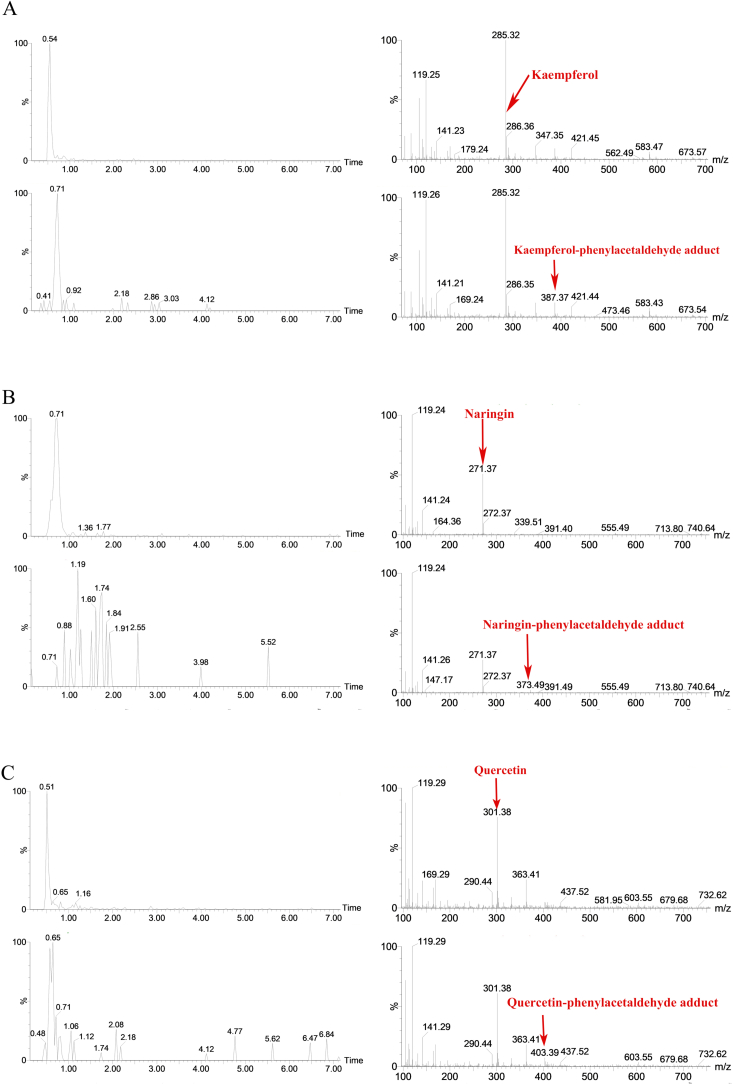
The mass spectrum of kaempferol and kaempferol-phenylacetaldehyde (A), naringin and naringin-phenylacetaldehyde (B), quercetin and quercetin-phenylacetaldehyde (C) adducts.

**Fig. 5:**
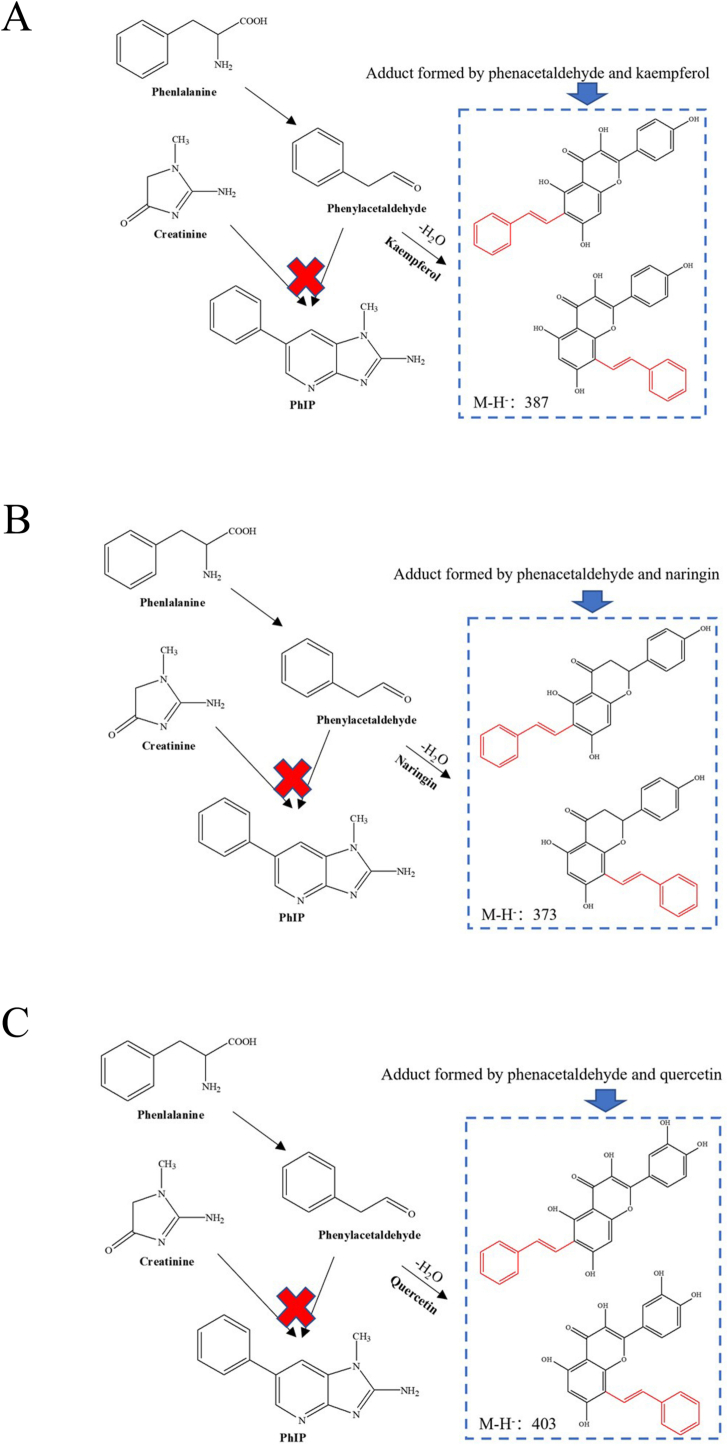
Postulated pathways for the inhibitory activity of kaempferol (A), naringin (B) and quercetin (C) against PhIP formation.

To further investigate the probable mechanism of phenylacetaldehyde elimination by kaempferol, naringenin, and quercetin, the possible compounds formed by phenylacetaldehyde reaction with them were identified by UPLC-MS analysis. The total ion chromatogram of the adduct of phenylacetaldehyde with kaempferol is shown in Fig. 4. Two ion peaks at *m*/*z* 387.37 [M-H]^−^ were detected by cationic ESI-MS, and the corresponding molecular weight was 386, which was consistent with the molecular weight of one molecule of kaempferol and one molecule of phenylacetaldehyde combined and one molecule of water removed. Similarly, for naringenin and quercetin, in the 372 and 402 cationic ESI-MS, two major ion peaks appeared at m/z 373.49 [M-H]- and M /z 403.39 [M-H]^−^, which are comparable to the molecular weights of naringenin and quercetin, respectively, when they bind to phenylacetaldehyde and then remove one molecule of water. A suggested collision process between polyphenols and the product ions of phenylacetaldehyde was examined, confirming the cause of the reverse Diels-Alder breakage of the C ring according to the previous study (Zhou et al., 2018). Based on the molecular weight of the three polyphenols, it is concluded that they can capture phenylacetaldehyde through electrophilic aromatic substitution and then eliminate water molecules, thereby inhibiting PhIP formation. Based on the structural properties of these three polyphenols, the condensation reaction pathways of phenylacetaldehyde with them were hypothesized. The structures of adducts are inferred in the way as depicted in Fig. 5. Because it has previously been shown that active carbonyl compounds (formaldehyde, phenylacetaldehyde, methylglyoxal, and glyoxal) can be conjugated and electrophilic substitution reactions occur at the 6 or 8 positions of the polyphenol A ring of the dihydroxyl structure to remove phenylacetaldehyde (Shakoor, Zhang, Xie, & Yang, 2022). As can be seen from Fig. 5, the two ion peaks may correspond to the electrophilic substitution products of C-6 and C-8 in the A ring. Phenylalanine was degraded by Strecker to produce phenylacetaldehyde, and the adduction produced by the reaction of phenylacetaldehyde with these three polyphenols with dihydroxyl structure was captured to reduce the formation of PhIP. Therefore, C-6 and C-8 are the active sites for kaempferol, naringenin, and quercetin to capture phenylacetaldehyde. Kaempferol, naringenin, and quercetin inhibit PhIP formation based on the mechanism of direct reaction with phenylacetaldehyde to form adduction. In addition, Zhao et al. (Zhou et al., 2022) have isolated, identified, and synthesized quercetin phenylacetaldehyde adduct as 8-C-(*E*-Phenylethenyl) quercetin (8-CEPQ) and 6-C-(*E*-phenylethenyl) quercetin (6-CEPQ). In this study, only mass spectrometry was used to investigate the existence of this compound. However, in order to more accurately confirm the existence of the adduct, it is necessary to prepare the standard of the compound in the chemical model system and further identify the compound with nuclear magnetic resonance technology.

## 4 Conclusion

This study used a chemical model system to analyze the structure-activity relationship and potential sites of action of polyphenols containing *m*-dihydroxyl structure. Results showed that the formation of PhIP was inhibited in a dose-dependent manner by polyphenols containing *m*-dihydroxyl structure. Furthermore, the inhibitory action of polyphenol containing *m*-dihydroxyl structure on PhIP production due to the presence of a hydroxyl group in the C ring's 3-position. The formation inhibition of PhIP will be significantly enhanced if the hydroxyl group simultaneously occupies the 3′-position of the B ring and the C ring. Hence, it is inferred that polyphenols containing *m*-dihydroxyl structure (kaempferol, naringenin, and quercetin) could capture phenylacetaldehyde to produce adduct by electrophilic aromatic substitution, eliminating water molecules and therefore inhibiting PhIP production. This study could provide more scientific theoretical and methodological reference for the quality control of HAAs in hot processed meat products and the application of natural extracts rich in polyphenols.

## CRediT authorship contribution statement

**Hao Dong:** Writing – review & editing, Supervision, Resources, Project administration, Methodology, Funding acquisition. **Qi Chen:** Writing – original draft, Validation, Software, Resources, Investigation, Formal analysis, Data curation. **Yan Xu:** Software, Resources, Methodology, Investigation, Data curation. **Chao Li:** Supervision, Project administration, Methodology, Funding acquisition. **Weidong Bai:** Visualization, Validation, Supervision, Resources, Methodology. **Xiaofang Zeng:** Visualization, Supervision, Funding acquisition, Conceptualization. **Qingping Wu:** Funding acquisition, Project administration, Supervision, Validation. **Huan Xu:** Data curation, Formal analysis, Investigation, Project administration, Resources. **Jinhua Deng:** Project administration, Supervision, Visualization.

## Declaration of competing interest

The authors declare that they have no known competing financial interests or personal relationships that could have appeared to influence the work reported in this paper.

## Data Availability

Data will be made available on request.

## Appendix A Supplementary data

Supplementary material Image 10
