# Supplementary material for: Effect and mechanism of polyphenols containing m-dihydroxyl structure on 2-amino-1-methyl-6-phenylimidazole [4, 5-b] pyridine (PhIP) formation in chemical models and roast pork patties
Source: Food Chem X. 2024 Jul 18;23:101672. doi: 10.1016/j.fochx.2024.101672 (PMC11321440; doi:10.1016/j.fochx.2024.101672)

**Effect and mechanism of polyphenols containing *m*-dihydroxyl structure on 2-amino-1-methyl-6-phenylimidazole [4, 5-b] pyridine (PhIP) formation in chemical models and roast pork patties**

Hao Dong^1,2^*, Qi Chen^2^, Yan Xu^2^, Chao Li^1^*, Weidong Bai^2^, Xiaofang Zeng^2^, Qingping Wu^3,4^*, Huan Xu^4^, Jinhua Deng^4^

^1^ Guangdong Province Key Laboratory for Green Processing of Natural Products and Product Safety, South China University of Technology, Guangzhou 510640, China

^2^College of Light Industry and Food Sciences, Zhongkai University of Agriculture and Engineering, Guangzhou 510225, China

^3^Guangdong Provincial Key Laboratory of Microbial Safety and Health, State Key Laboratory of Applied Microbiology Southern China, Key Laboratory of Microbiomics and Precision Application, Ministry of Agriculture and Rural Affairs, Institute of Microbiology, Guangdong Academy of Sciences, Guangzhou 510070, China

^4^Guangdong Huankai Microbiology Science & Technology Co., Ltd, Guangzhou 510700, China

*Corresponding authors: Chao Li ([felichao@scut.edu.cn](mailto:felichao@scut.edu.cn)); Qingping Wu (wuqp203@163.com)

Tel: +86-20-89003827; Fax: +86-20-89003827

Figure S1 The PhIP production pathway (A) and the inferred mechanism of polyphenols' suppression of PhIP production (B).


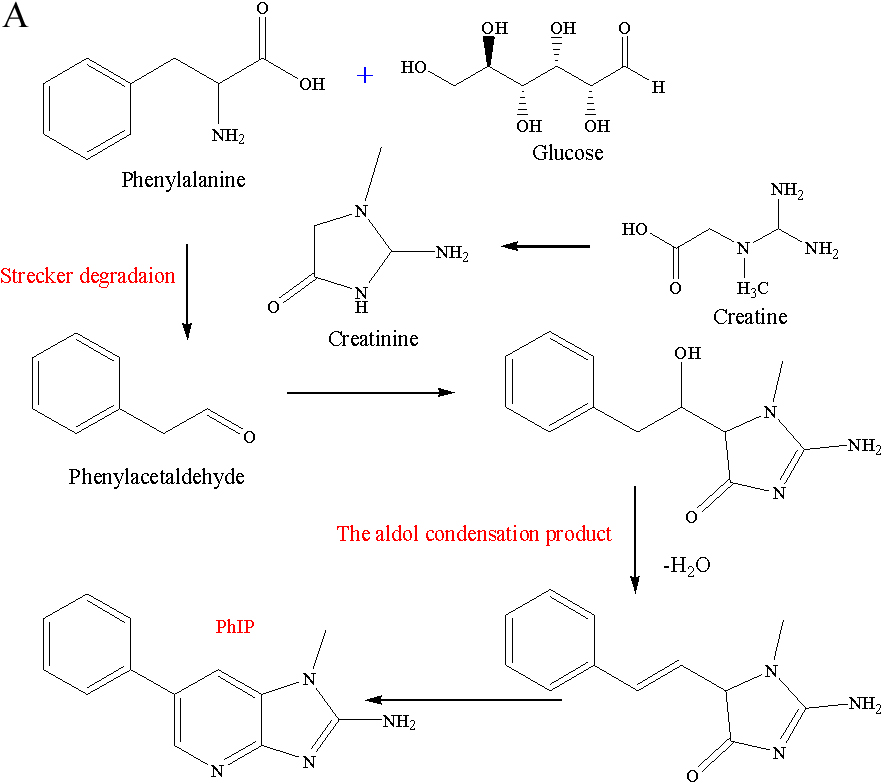


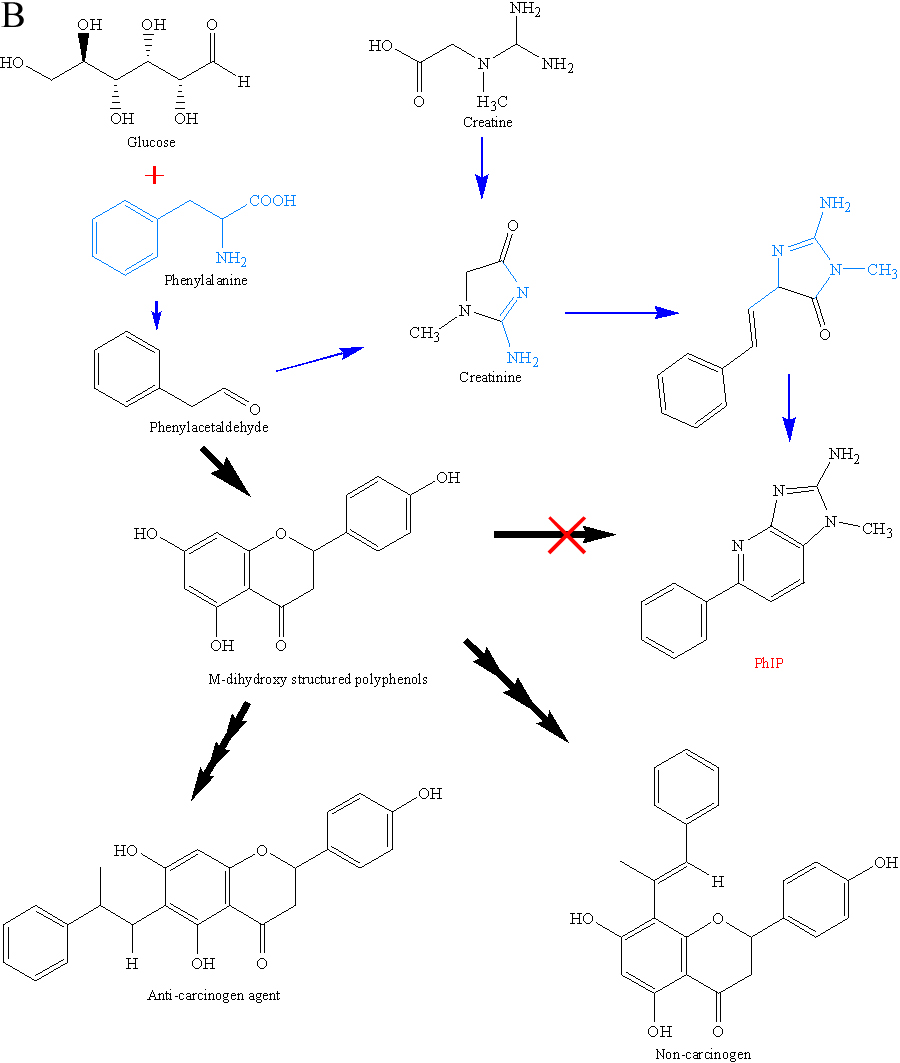


Figure S2 (A): UV absorption spectra of blank, kaempferol, naringin and quercetin after 200 min of heating at 220 °C;

(B-G): MS/MS chromatograms demonstrating relative abundance of creatinine, creatine, diethylene glycol, phenylalanine, glucose, phenylacetaldehyde: (B) m/z 114, (C) m/z 130, (D) m/z 107, (E) m/z 164, (F) m/z 203, and (G) m/z 119 in chemical models at 220 °C for 200 min.


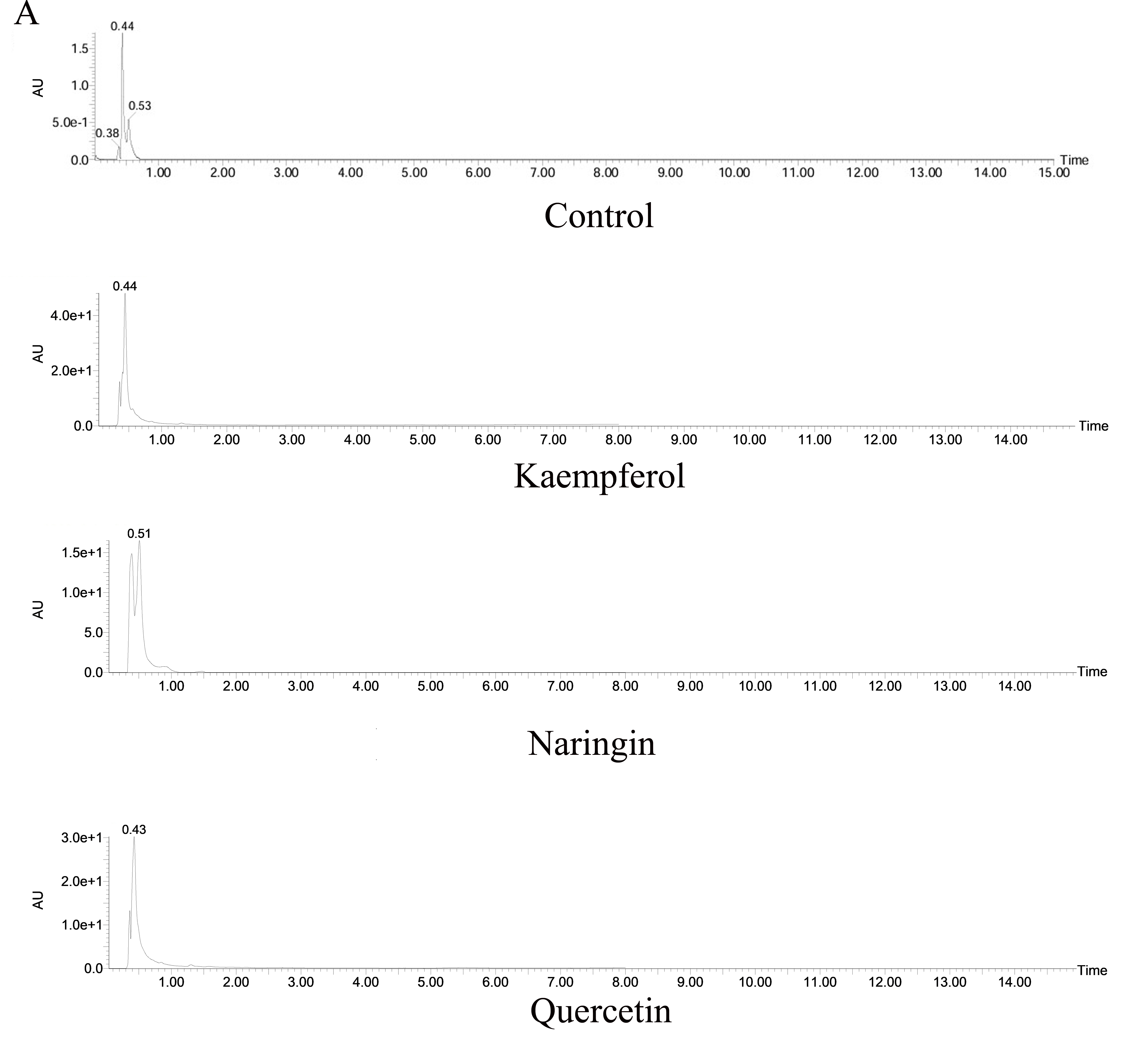


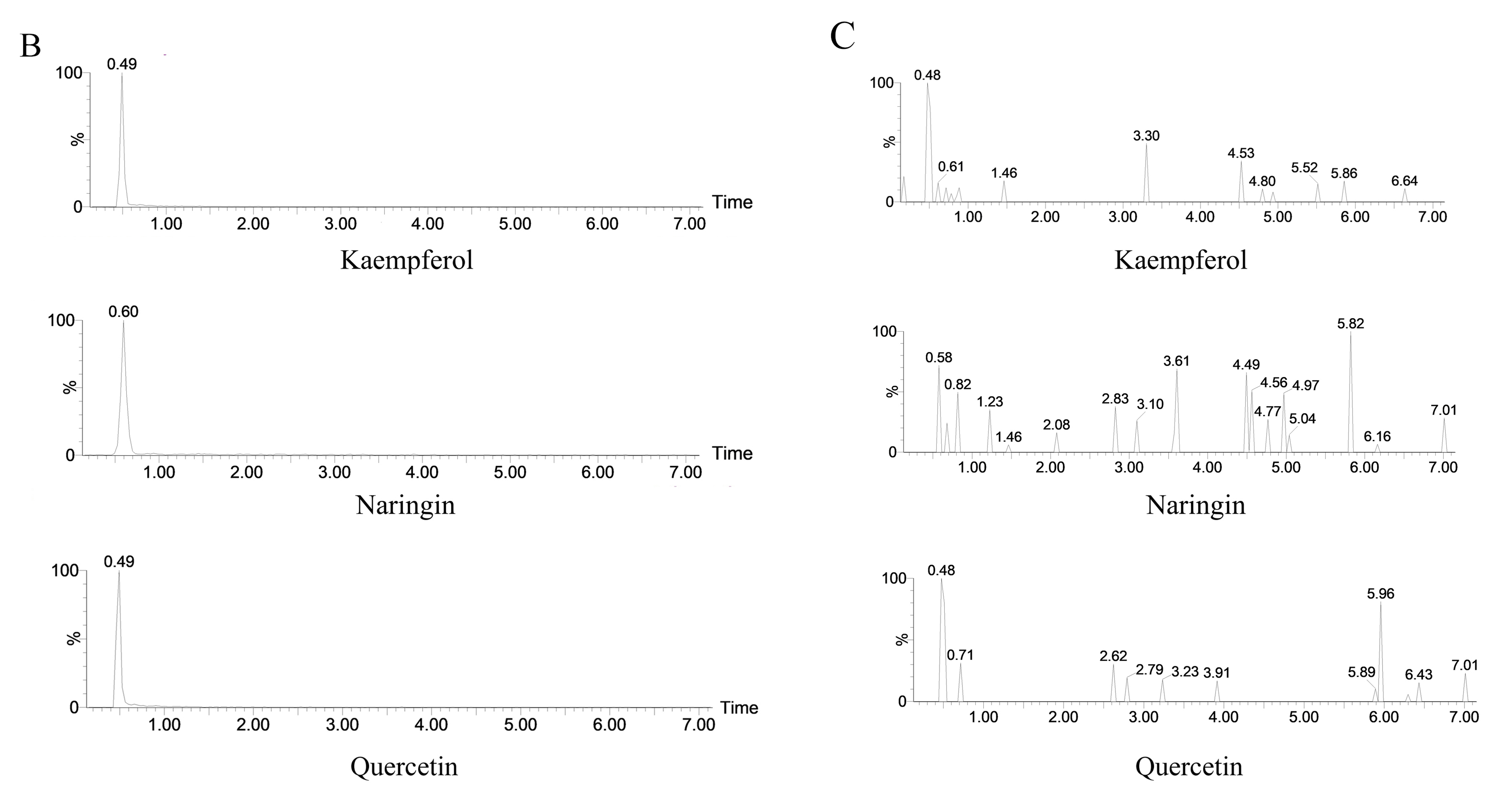


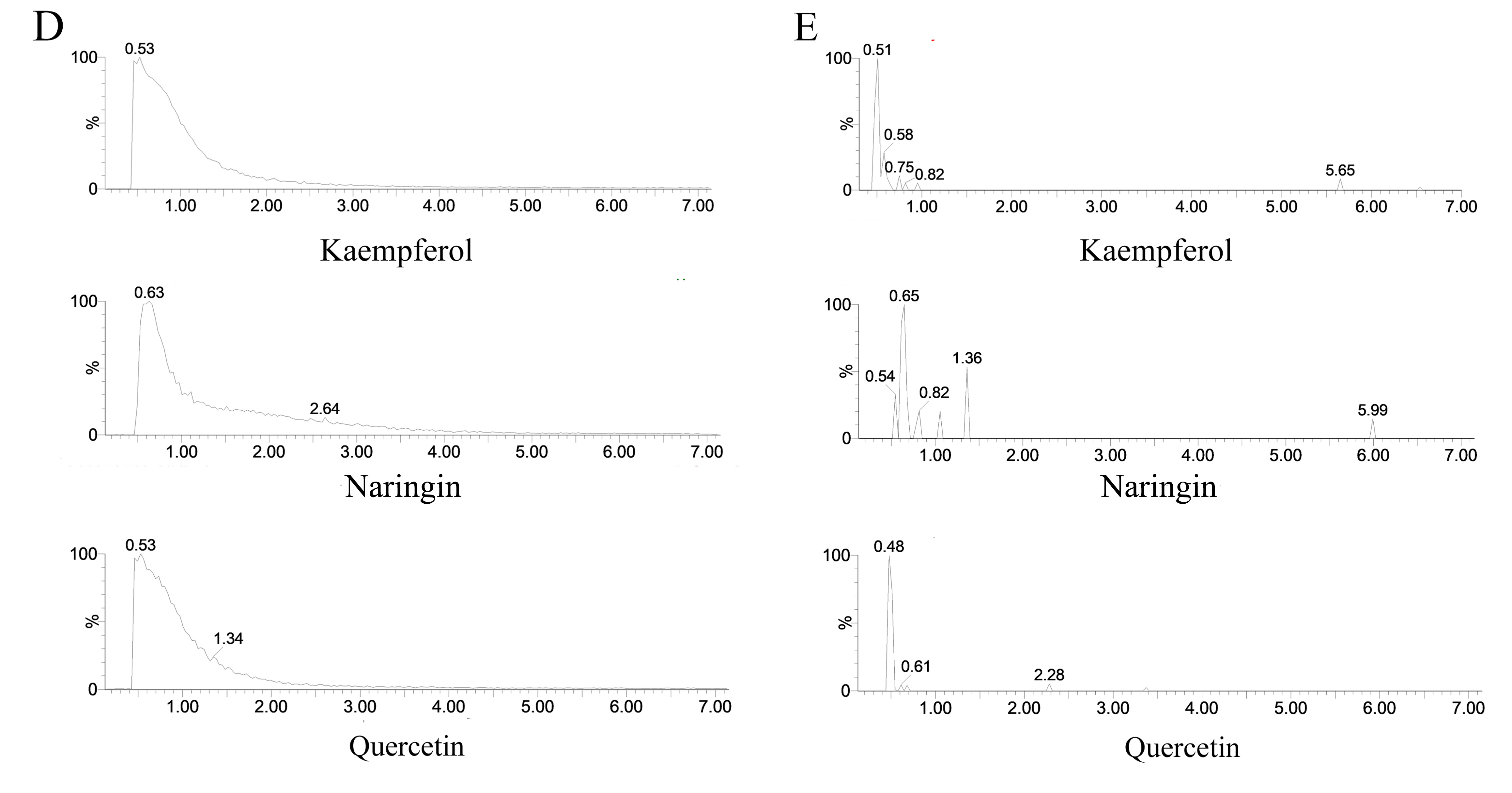


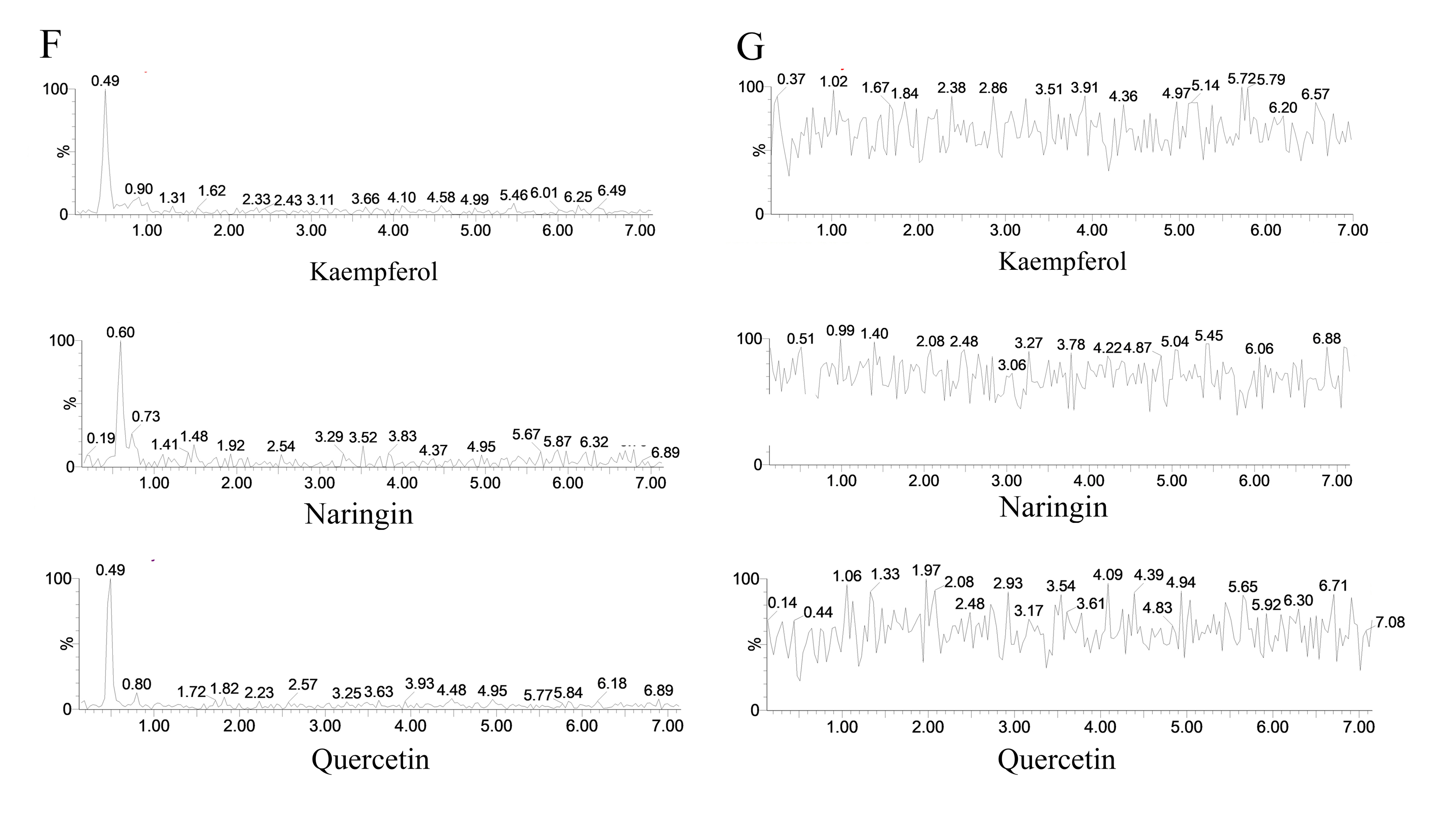

Supplement: Supplementary file 1 — Supplementary material [file mmc1.docx]
